# Human menstrual blood: a renewable and sustainable source of stem cells for regenerative medicine

**DOI:** 10.1186/s13287-018-1067-y

**Published:** 2018-11-21

**Authors:** Haining Lv, Yali Hu, Zhanfeng Cui, Huidong Jia

**Affiliations:** 1 Department of Obstetrics and Gynecology, Nanjing Drum Tower Hospital, Graduate School of Peking Union Medical College, 321 Zhongshan Road, Nanjing, China; 2 Tissue Engineering Group, Institute of Biomedical Engineering, Department of Engineering Science, University of Oxford, ORCRB, Roosevelt Drive, Headington, Oxford, OX3 7DQ UK

**Keywords:** Menstrual blood, Menstrual blood-derived stem cells, Regenerative medicine

## Abstract

Stem cells (SCs) play an important role in autologous and even allogenic applications. Menstrual blood discharge has been identified as a valuable source of SCs which are referred to as menstrual blood-derived stem cells (MenSCs). Compared to SCs from bone marrow and adipose tissues, MenSCs come from body discharge and obtaining them is non-invasive to the body, they are easy to collect, and there are no ethical concerns. There is, hence, a growing interest in the functions of MenSCs and their potential applications in regenerative medicine. This review presents recent progress in research into MenSCs and their potential application. Clinical indications of using MenSCs for various regenerative medicine applications are emphasized, and future research is recommended to accelerate clinical applications of MenSCs.

## Background

The endometrium undergoes more than 400 cycles of regeneration, differentiation, and shedding over the whole reproductive period of a woman. Human endometrial stem cells play an important role in this cyclic regeneration and repair. Endometrial stem cells (EndoSCs), including epithelial, stromal, and endothelial cells, may contribute to the periodic endometrial regeneration [1], mainly reside in the perivascular region of both the basalis and functionalis of the endometrium [2]. When exfoliated in the menstrual blood, these EndoSCs are hence referred to as the menstrual blood-derived stem cells (MenSCs) [3]. The advantages of MenSCs include non-invasiveness of extraction, high proliferation ability, and short doubling time, and maintenance of chromosome karyotyping after up to 68 generations, which qualifies MenSCs as an ideal source of regenerative cells desperately needed for transplantation, neurological disorders, and cancer therapy, etc. [4, 5].

## Cellular features of the endometrium and types of MenSCs

The endometrium, which consists of luminal epithelium, glandular epithelium, and an extensively vascularized stroma, structurally and functionally falls into two compartments, viz, functionalis and basalis [3]. Endometrial glands are lined with pseudo-stratified columnar epithelium extending from the luminal epithelium to the endometrial/myometrial junction. The functionalis consists of the upper two thirds of the glands surrounded by loose vascularized stroma. Being a germinal supplier for new functionalis replacement in each cycle, the basalis is composed of the lower one thirds of glands, stroma, and large vessels [6]. Gargett et al. considered that human endometrial stem cells include epithelial progenitor cells, endometrial mesenchymal stem cells (eMSCs), and endothelial progenitor cells [3], while Evans et al. characterized endometrium-specific stem cells into epithelial progenitor cells, side population (SP) cells, and eMSCs [7].

### Endometrial epithelial progenitor cells

Within the first 48 h of menses, with stumps of the gland remaining in the basalis, a rapid repair and re-epithelization of the endometrium lining occurs to cover the exposed basal surface. Epithelial progenitor cell populations locate within the residual glands of the basalis [8]. Evidence was provided by the presence of colony-forming units (CFUs) in suspension cells from hysterectomy specimens [6]. These large single cell-derived epithelial CFUs have high proliferative potential and can differentiate into large glandular-like structures in 3D culture [9]. Although pluripotent stem cells can be isolated from endometrial biopsies or menstrual blood, epithelial progenitor cells cannot be obtained from menstrual blood, either because they are not present in the menstrual blood or because they are simply eclipsed by the huge amount of stromal fibroblast populations [10]. Previous study has implied that the niche of epithelial progenitor cells is more likely to be in the basal layer than in the functional layer [3, 6]. In fact, epithelial progenitor cells have also been identified in the endometrial basal layer of post-menopausal women, suggesting that they may serve as a source of post-menopausal endometrial stem cells [11].

Stage-specific embryonic antigen (SSEA)-1 is the most abundant stem cell marker found in endometrial basal glandular epithelial cells from hysterectomy tissues of women [12]. Compared with SSEA-1^−^ cells, SSEA-1^+^ epithelial cells have significantly greater telomerase activity and longer mean telomeres, as well as more pronounced quiescence and lower proliferation rates, which are the hallmarks of epithelial progenitor cell populations. Human endometrial epithelial progenitor cells may be a subset of the SSEA-1^+^ population, located in the functionalis adjoining the basalis [6]. Leucine-rich repeat-containing G-protein-coupled receptor (LGR5) has also been detected on the rare epithelial cells in the lower functionalis adjacent to the basalis [13]. However, the small population of endometrial LGR5^+^ cells has a confined capacity to form an endometrium-like structure which appears to have characteristics of resident macrophages at the perivascular microenvironment [14]. Since macrophages are also known to exhibit stem cell properties to regulate self-replication and tissue repair [15], LGR5 could be a marker to identify macrophages during tissue repair but not to specify epithelial progenitor cells. N-cadherin can be used to identify and isolate clonogenic and self-renewing human endometrial epithelial progenitor cells [16]. This type of cell possesses the proliferative potential to be differentiated into cytokeratin^+^ gland-like structures and to locate these gland-associated cells in the basalis abutting the myometrium. This finding indicates this molecule could be a novel marker for recognizing the more primitive progenitor cells that differentiate from the basalis of the endometrium.

### eMSCs and MenSCs

The identification of eMSCs is a group of small and compact mesenchymal stem cells (MSCs) or colony-forming unit fibroblasts (CFU-F) [17]. They have perivasculature location within both the functionalis and basalis and are also detected in shedding fragments in menstrual blood [9]. Co-expression of CD140b and CD146 is considered as a pericyte-specific property, which was used in identifying the eMSCs location in human endometrium by Darzi and colleagues. Meanwhile, a single antibody W5C5, which recognizes epitope Sushi-domain-containing-2 (SUSD2) on eMSC, was used to sort out a clonogenic, multipotent, and self-renewing cells population [17]. This discovery indicates that perivascular markers CD146, platelet-derived growth factor receptor (PDGFR)-β, and SUSD2 can be used as specific markers for isolation of MenSCs because eMSCs are the major components of MenSCs obtained during menstrual shedding [18]. The cellular composition of MenSCs can be distinguished by CD117^−^ stromal fibroblasts and highly proliferative CD117^+^ SUSD2^+^ eMSCs [6, 10]. Viable stromal cells obtained from menstrual blood presented MenSC profile of plastic adherence, multi-lineage differentiation potential, expression of classical MSCs surface markers, and stable karyotype in culture. However, without selective marker enrichment, as is currently achieved for endometrial biopsy-derived eMSCs, they may not be as consistent or as efficacious as eMSCs [17].

## Cell markers of MenSCs

Human MenSCs are positive for CD29, CD44, CD73, CD90, CD105, and CD166, while negative for CD14, CD34, CD38, CD45, and CD117. Weakly expressing human leukocyte antigen (HLA)-ABC [19] and expressing no HLA-DR indicate the low immunogenicity of the MenSCs [3, 20, 21]. Co-expression of CD140b and CD146 has been used to isolate eMSCs [22] and MenSCs [17] and can be discriminated from CD146^−^CD140b^+^ endometrial stromal fibroblasts and CD146^+^CD140b^−^ endometrial endothelial cells [23]. MenSCs also express human telomerase reverse transcriptase (hTERT) and display telomerase activity [24, 25]. Other markers such as SSEA-4, NANOG, and sex-determining region Y-box2 (SOX2) [26, 27] were documented by Borlongan et.al. who made the point that the salient feature of MenSCs to present different stem cell marker profiles in an environment-dependent manner would perfectly fulfill the requirement for tailored tissue transplantation [28]. This may explain the discrepancy of reported SSEA-4 expression of MenSCs ranging from 0 [29] to 19.4% [4] and even higher [30]. Other factors of donor’s conditions such as age, use of contraception, and environmental factors are speculated to contribute to this disparity too.

Expression of the embryonic stem cell surface marker octamer-binding transcription factor 4 (OCT-4), which is commonly used to identify MenSCs, is not subjected to the cyclical alteration of the endometrium. It has two protein isoforms. OCT-4A is a transcription factor in the nucleus that maintains the differential potency of embryonic and adult stem cells, whereas cytoplasmic expressed OCT-4B is not involved in stem cell regulation [31]. The cytoplasmic localization of OCT-4 detected in MenSCs therefore led to doubts about the pluripotency of MenSCs [6, 28, 32]. However, a recent investigation of mouse endometrium revealed the presence of very small embryonic-like stem cells (VSELs), which expressed nuclear transcripts of OCT-4, accounting for approximately 0.069% of the total endometrial cells. The same authors termed the relatively larger and more abundant progenitor cells expressing cytoplasmic OCT-4 as endometrial stem cells [33]. It is speculated that the importance of nuclear OCT-4 for VSELs, the primordial germ cells, or their precursors to differentiate into tissue-specific cell types is gradually attenuated over the period of differentiation, during which OCT-4 translocates from the nucleus into the cytoplasm [34].

Perivascular sushi domain containing-2 (SUSD2) is another useful molecule to specify multipotent and self-renewal MenSCs. SUSD2^+^ cells represent 4.2% of the stromal cell population, which encloses the majority of the clonogenic population in endometium [17].

## Collection and culture of MenSCs

On the second day following menstruation initiation, a menstrual cup can be used to collect menstrual blood specimens from healthy donors. Sample blood is then mixed with an equal volume of phosphate-buffered saline (PBS) and the supplementations listed in Table 1. Mixed samples can be stored at 4 °C for up to 3 days without influencing MenSC activities [26]. Ficoll paque is added slowly to the mixture, followed by centrifugation for 10 min. The middle white layer of endometrial cells is transferred into growth medium (ingredients are listed in Table 2). The cell pellets are subsequently re-suspended in growth medium. After 72 h incubation, the non-adherent cells are discarded, leaving the attached adherent cells for further culture. The isolated MenSCs should display classical cellular characteristics of MSCs, i.e., spindle-shape, elongated, or clustered fibroblastic morphology, with clearly visible nuclei.

**Table 1:** Supplements in PBS for MenSC separation

	Supplements				Reference
	Amphotericin B	Streptomycin	Penicillin	EDTA-Na2	
Protocol 1	40 mL/L (Sigma)	40 mL/L (Sigma)	40 mL/L (Sigma)	20 ml/L	[25]
Protocol 2	2.5 mg/L (Gibco)	100 mg/L (Sigma)	100 U/mL (Sigma)		[20]
Protocol 3	250 mg/L	100 mg/L	100 U/mL	2 mM	[26]

**Table 2:** Growth medium for MenSCs

	Components					Reference
	Base	FBS (*v*/*v*)	Antibiotics	Other nutrients	Growth factors	
Protocal 1	DMEM	20%	1% penicillin and streptomycin	1% glutamine		[25]
Protocal 2	DMEM/F12	10%	100 U/mL penicillin and 100 mg/mL streptomycin			[20, 26]
Protocal 3	Basic MSU-1	5%		2 mM *N*-acetyl-l-cysteine, 0.2 mM l-ascorbic acid-2-phosphate		[74]
Protocal 4	Minimal alpha GutaMAX	10%	10,000 U/mL penicillin, 10 mg/mL streptomycin and 25 μg/mL amphotericin in 0.9%NaCl	20 mmol/L *N*-2-hydroxyethylpiperazine-*N*-2-ethane sulfonic acid pH 7.2–7.4	1.6 nmol/mL prednisolone sodium phosphate 80 μU/mL insulin 20 ng/mL FGF	[75]

## Comparison of MenSCs with MSCs from other sources

### Morphology

Adipose-derived MSCs (ADSCs) and MenSCs have the greatest inter-cell contact inhibition which presents an enhancing trend towards higher passage numbers. Increasing passaging number also resulted in differentiation of ADSCs and umbilical cord-derived cells MSCs (UCMSCs) into polygonal shapes with elongated protrusions, in contrast to the remarkably granular protrusions on the surface of MenSCs [35]. Compared with BMSCs, freshly isolated MenSCs are more plastic adherent [36] and their morphology mainly depends on their density. When the amount of MenSCs per square centimeter exceeded 500, they became small and flat, while the BMSCs retained a long and large fibroblastic shape [37]. In agreement with this observation, Ren et al. revealed a phenomenon of flattening and fragmentation in MenSCs along the passaging [38], whereas polygonalization and cytoplasmic granulation occurred in UCMSCs, leaving only dental pulp-derived stem cells (DPMSCs) to retain the initial fibroblastic morphology [39]. Human amniotic fluid mesenchymal stem cells (AFMSCs) which possess a multilineage differentiation potential are present in the amniotic fluid. They are highly proliferative with a normal karyotype and fibroblastic morphology after long-term in vitro culture and do not form teratomas when transplanted in vivo [40]. Protocol for isolating different types of MSCs is listed in Table 3.

**Table 3:** Protocol for isolating different types of MSCs

Cell types	Protocol for isolating different types of MSCs	
MenSCs	Menstrual blood (5 mL) from the second day of normal menstrual period was collected with a menstrual cup and transferred into PBS containing supplementations which are listed in Table 1. The menstrual blood was diluted using the same volume of aseptic PBS. MenSCs were separated by lymphocyte separation medium with a density gradient centrifugation. The cells were inoculated in growth medium as shown in Table 2 and cultured at 37 °C with 5% CO2 and saturated humidity. The medium was changed every 3 to 4 days according to the growth of the cells.	[49]
AFMSCs	Two or 3 mL of amniotic fluid was obtained from pregnant women during routine amniocentesis at 16–18 weeks of gestation, and cells were immediately isolated by centrifugation. The supernatant was discarded, and the cell pellet was suspended in standard medium composed of low-glucose DMEM and other supplementations and incubated at 37 °C with 5% CO2 in a humidified atmosphere. The medium was changed every 3 to 4 days according to the growth of the cells.	[76]
BMSCs	The bone marrow cells were diluted in an equal volume of PBS, isolated with Ficoll, and then centrifuged. The cells were then rinsed with PBS and cultured in DMEM containing 10% FBS at 37 °C in a humidified incubator infused with 5% CO2. Adherent cells were collected after 5 days.	[77]
UCMSCs	The umbilical cords were rinsed with PBS in penicillin and streptomycin, and then the umbilical arteries and veins were removed. The remaining tissue was cut into 1 to 2 mm pieces and floated in DMEM/F12. The pieces were subsequently digested in an enzyme cocktail for 3 h at 37 °C. After this tissue was crushed with forceps and large pieces were removed, human umbilical cord MSCs were harvested and plated into a culture flask. The cells were incubated at 37 °C in an incubator with 5% CO2 at saturating humidity. The medium was changed every 3 to 4 days according to the growth of the cells.	[39]
ADSCs	ADSCs were obtained by adipose tissue (2–3 mL) digestion with collagenase A (Sigma Aldrich, Milan, Italy) and then seeded onto a T25 flask at 37 °C, 5% CO2 in DMEM containing 10% FBS and antibiotics to select adherent cells. ADSC cell lines at passages 3–6 were used for the experiments.	[78]
DPMSCs	Human dental pulp was extracted from third molar or permanent teeth of adult subjects (18 and 35 years of age) after informed consent of patients undergoing routine extractions. Dental pulp was removed from the teeth and then immersed in a digestive solution (3 mg/mL type I collagenase plus 4 mg/mL dispase in α-MEM) for 1 h at 37 °C. Once digested, the pulp was dissociated and then filtered onto Falcon Cell Strainers to obtain a cell suspension. Cells were then plated in 25 cm^2^ flasks and cultured in culture medium at 37 °C and 5% CO2. Cells obtained from a single dental pulp were plated at clonal density (1.6 cells/cm^2^). After 6 days of culture, eight cell populations were isolated from nodules originated by single cells.	[79]

### Proliferative capacity

The cell doubling time of MSCs follow the order of UCMSCs (21 h) < MenSCs (26 h) < ADSCs (30 h), with MenSCs right in the middle [35]. Another study suggested that MenSCs may have better proliferative capability with a shorter population doubling time of 20 h [5]. The growth curve of MenSCs showed a slow increase at the early stage which peaked at the eighth passage before gradually slowing down. After slow proliferation in the first 2 days, UCMSCs, PMSCs, and MenSCs entered their rapid logarithmic growth phases with a duration of 4–5 days, which then plateaued on days 6–7 [38]. The expansion potential of AFMSCs is greater than BMSCs [40], but its comparison with MenSCs has not been reported till now. Trend of proliferation capacity of different MSCs can be summarized as UCMSCs > DPMSCs > MenSCs > ADSCs > BMSCs.

### Surface markers

MenSCs express OCT-4, whereas BMSCs do not [36–38, 41]. Unlike with other MSCs, expression of MSCs markers, CD29, CD44, and CD90 in MenSCs reduced to less than 95% when the cultured cells reached P10, indicating a declined stemness activity of MenSCs upon more passaging. CD44, the important adhesion molecule associated with cell migration, is 10 times higher in MenSCs than in BMSCs and UCMSCs indicating that MenSCs may have a stronger adhesion ability [35]. Surface marker phenotypes of different human stem/stromal cell populations are shown in Table 4.

**Table 4:** Surface marker phenotypes of different human stem/stromal cell populations

Markers	Cell type					
	MenSCs	AFMSCs	BMSCs	UCMSCs	ADSCs	DPMSCs
CD13		+	+			+
CD14	–	–	–	–	–	–
CD29	+	+	+	+	+	+
CD31	–	–	–	–	–	–
CD34	–	–	–	–	–	–
CD38	–		–			
CD44	+	+	+	+	+	+
CD45	+	–	–	–	–	–
CD56						+
CD61						+
CD73	+	+	+	+	+	+
CD79-α or CD19	–	–	–	–	–	–
CD90	+	+	+	+	+	+
CD105	+	+/−	+	+	+	+
CD117	–	–	–			
CD144		–	–			
CD146	+	+	+			
CD166	+	+	+			+
HLA-ABC	+/−	+	+			+
HLA-DR	–	–	–	–	–	–
Stro-1	+	+	+	+	+	+
OCT-4		+/−	+			
SSEA-4	+	+	+			
Sox-2	+	+	+			
h-TERT	+	+	+			
SUSD-2	+					
NANOG	+					
N-cadherin	+					
Reference	[3, 16, 17, 19–21, 24, 25, 80–82]	[76, 80–82]	[76, 80–82]	[76, 80–82]	[76, 80–82]	[76, 80–82]

### Multi-differentiation potential

UCMSCs, DPMSCs, and MenSCs all display osteogenic and adipogenic differentiation to various extents [38]. MenSCs have the lowest potential to be differentiated into adipocytes while the osteoblastic differentiation ability among these three MSCs is rather similar. The differentiation potential of chondrogenesis and osteogenesis between MenSCs and BMSCs had no difference [36]. However, one of the osteocyte specific markers alkaline phosphatase, a crucial player in bone mineralization, was less upregulated in differentiated MenSCs than in differentiated BMSCs [37]. Interestingly, this inferiority in osteogenic differentiation can be successfully overcome by substitution of human platelet lysate with FBS. MenSCs were more able to be specialized into cardiomyocytes than BMSCs [41]. Although the ability of MenSCs to differentiate into neural cells was inferior to BMSCs, the levels of functional calcium, potassium, and sodium channel genes in MenSCs during the process of differentiation were significantly higher than those in BMSCs [42].

## Clinical application of MenSCs

As MenSCs have not only robust expression of the pluripotent and embryonic stem cell surface markers OCT-4, SSEA-4, and c-kit, but also hTERT and high telomerase activity, their application in regenerative medicine has already emerged as a fertile field. Apart from that, MenSCs can be stably and effectively infected with lentiviruses, suggesting that the cells could serve as a valuable tool for gene delivery. The expression of matrix metalloproteins and large amounts of angiogenetic and antiapoptotic cytokine secretion firmly support the advantages of MenSCs in vascular remodeling, tissue repair, and regeneration [25, 43, 44]. Also, it had little risk of tumorigenicity [26]. On top of it, MenSCs are shown to exert an immunoregulatory role, especially immunosuppression, by inhibiting the expression of proinflammatory factors, such as tumor necrosis factor-α, interferon-γ, and interleukin (IL)-6, promoting the secretion of IL-4 and increasing the number of regulatory T cells and M2 macrophages [45].

Based on a wide array of overall benefits of MenSCs, it has provided a desirable medical tool for multiple-lineage differentiation into adipocytes, osteoblasts, chondroblasts [4, 10, 25, 37] and skeletal cells, cardiomyocytes, and neural cells [46, 47], projecting profound implications onto a great number of human diseases, not to be confined as only a new hotspot in basic research.

### Reproductive system

Using the electrocoagulation method, Zhang et al. established a mouse model to simulate intrauterine adhesions (IUA) [48]. Mice synchronized at the secretory phase were injured, and human MenSCs were injected via tail veins. A more remarkable recovery of the endometrium was observed in mice with MenSCs engraft, shown by an increase in endometrial thickness and the density of the micro-vessel density. The restoration of the endometrium was functionally confirmed by improved fertility of the MenSCs-treated mice. Zheng et al. found that MenSCs can differentiate into endometrial cells both in vitro and in vivo, but MenSCs from patients with severe IUA had poorer clonogenecity and decreased OCT-4 expression, compared to that from healthy subjects [49]. In a pilot clinical trial using MenSCs in the treatment of IUA, Tan et al. transplanted autologous MenSCs into seven patients with severe IUA [50]. Among them, the endometrial thickness of five patients significantly increased to 7 mm. The encouraging outcome included one spontaneous pregnancy and two successful conceptions out of the remaining four patients who received embryo implantation. Although there are still some concerns and limitations to the results, autologous MenSC transplantation is surely worth more comprehensive exploration. For premature ovarian failure (POF), the Liu team introduced MenSC transplantation in bilateral ovaries to treat cyclophosphamide-induced mouse models of POF and found that MenSCs survived at least 14 days in vivo, leading to elevated expression of ovarian granulosa cell markers anti Müllerian hormone, inhibin α/β and follicle-stimulating hormone receptor, and the proliferation marker Ki67 [51]. Further mechanistic analysis demonstrated differentiation of MenSCs into ovarian tissue-like cells under the microenvironmental influence in the POF mice, thereby restoring ovarian function. Using similar POF mouse model and green fluorescent protein-labeling, Lai et al. located the injected MenSCs in the ovarian stroma in the mice, which had weight gain, improved estrus cyclicity, and restored ovarian functions and protection from the exhaustion of the germline stem cell pool [52]. Rajabi et al. deployed an in vitro 3D co-culture system using MenSCs and mouse preantral follicles [53]. Their results showed increased follicular growth indices and improved maturation parameters, resulting from higher secretion of 17 β-estrodiol and progesterone by follicular cells under MenSC induction. Other growth factors such as activin A, transforming growth factor-β, vascular endothelial growth factor, and fibroblast growth factor (FGF) may have also been involved.

At present, tissue engineering scaffolds using autologous MenSCs and synthetic mesh have been used to repair the defects in pelvic organ prolapse (POP) patients. In vitro stimulation using connective tissue growth factor can direct MenSC differentiation into cell types favoring POP repair, e.g., collagen-producing fibroblasts [54]. MenSCs can promote tissue vascularization, integration, collagen deposition, reduced chronic inflammatory response, increased fibers, and graft tolerance by immunosuppression [55].

### Cardiovascular system

Powerful cardiomyogenesis potential of MenSCs is evidenced by Hida et al. who harvested human MenSCs to test their use in treating myocardial infarction (MI) of rats [47]. When in vitro co-cultured with cardiomyocytes, almost half of MenSCs started strong beating after 5 days and approximately 27 to 32% of them were successfully differentiated into cardiac tropolin–I positive cardiomyocytes. After the cell suspension of MenSCs was then injected into the infarcted myocardium in rats, the MI area was significantly shrunken, where a mass of MenSCs was detected with abundant α-actinin and cardiac troponin expression. Similarly, Jiang et al. examined the benefits of MenSC transplantation by injecting MenSCs straight into five sites of ischemic border zone of infarct in an immunocompetent rat model [56]. The results demonstrated that MenSCs salvaged cardiac viability in the MI, increased myocardium volume, promoted endogenous cardiac regeneration, reduced apoptosis, and increased vascular density, mainly through paracrine effects exerted by MenSCs. But there was no evidence of MenSC differentiation directly into vascular cells or cardiomyocytes *per sei*. Another piece of supportive and rather longer-term evidence of transplantation of MenSCs in a patient with congestive heart failure was provided by Ichim et al. in a case report [57]. It was reported for the first time that, in this patient, increased ejection fraction and immensely improved heart failure score plus pro-BNP index were achieved 2 years after MenSC administration. It is worth noting that MenSCs are by some authors referred to as endometrial regenerative cells (ERCs) from human menstrual blood. Other clinical parameters also showed an optimistic all-round wellbeing of the patient without any sign of side effects from the ERCs administrations. The immunoregulatory role of MenSCs was elucidated by Lan et al. by assessing their efficacy to induce allograft tolerance in cardiac transplantation [58].

### Nervous system

MenSCs may provide sufficient cell replacement for stroke repair. By co-culturing MenSCs with primary rat neurons exposed to oxygen glucose deficiency in vitro or transplantation of MenSCs into a surgically induced ischemic stroke rat model in vivo, Borlongan et al. reported a significant neuroprotective role of MenSCs in hypoxic injury [28, 59]. Functional deficit resulted from stroke was also rescued by MenSC treatments, revealed by behavioral analysis of the rats administered with MenSCs either intracerebrally or intravenously. The secretion of cytokines essential for neuron protection and functions plays a more important role in the therapeutic mechanism of MenSCs than their neuron differentiation [44].

The Han team injected MenSCs intratumorally or intravenously into an aggressive glioma rat model and yielded significant tumor inhibition, indicated by decreased angiogenesis and CD133^+^ cell number, a signature of tumor cells residing in hypoxic niche [60]. The tropism of MenSCs for glioma also betokens its application in selective delivery of therapeutic agents in glioma treatment. Besides, MenSCs are a powerful vehicle for delivering genes or drugs to trigger tumor site-specific apoptosis [61].

### Digestive system

MenSCs may be a feasible way around the problems of hepatocyte transplantation in severe liver diseases [62]. Cultured in hepatogenic medium, MenSCs were firstly induced to differentiate into functional hepatocyte-like cells (HLCs) in vitro, followed by intrasplenical transplantation into 2/3 partial hepatectomy (PH) mice. The liver function of PH + HLCs mice was significantly recovered. Lu et al. reported another example of restoration of liver function by MenSCs [63]. MenSCs were injected into mice 30 min after the induction of acute liver injury (ALI), and the protective role of MenSCs was observed 24 h later, alongside, or maybe caused by enhanced anti-inflammatory cytokine secretion. Meanwhile, two studies performed by Xiang’s group [64, 65] also investigated the therapeutic benefits of MenSCs in liver repair. In one of their works [64], they pre-treated the mice suffering fulminant hepatic failure with MenSCs-derived exosomes (MenSCs-Ex), which have higher secretion of cytokines such as intercellular adhesion molecule 1, angiopoietin-2, insulin-like growth factor binding protein 1, osteoprotegerin, IL-6 and IL-8, than MenSCs. A much lower mortality was achieved in the MenSC-Ex mice than in the control, through reduced recruitment of liver mononuclear cells and pro-apoptosis caspase-3, resulted in significantly reduced hepatocellular necrosis. In their other study [65], they tested the antifibrotic effect of MenSCs in a chronic liver disease by a mouse model of CCl_4_-induced liver fibrosis. Two weeks after the transplantation, migrated MenSCs were detected at the injury site, the activation of stellate cells was dramatically counter-acted and liver function was significantly improved.

The anti-inflammatory modulation of MenSCs in liver injury was finely mirrored in a colitis mouse model [66], where MenSCs were injected three times consecutively in a mouse model of colitis. A similar impact of MenSCs on immunoregulation was displayed on day 14 following colitis induction, as shown by a reduced disease activity index, twisted immune factor profile to favor anti-inflammatory response, splenic inflammatory infiltration, and reduced MHC-II^+^ dendritic cells. The data proved the advantage of MenSCs to attenuate colitis development and progress [63].

Wu et al. intravenously injected human MenSCs into streptozotocin-induced diabetic mice and found ameliorated diabetic symptoms of the treated mice with expanded life span, reversed histological changes of islets, and better glycemic control [5]. It was concluded that MenSCs promoted endogenous regeneration of β-cell progenitors to recover the treptozotocin-induced pancreatic damage via a paracrine mechanism, instead of differentiating into insulin-producing cells directly [5, 67].

### Musculoskeletal system

In-depth investigation of Duchenne muscular dystrophy (DMD) undertaken by Umezawa’ s team [68, 69] not only successfully transdifferentiated MenSCs into myoblasts/myocyts in vitro, but also restored sarcolemmal expression of dystrophin in dystrophied muscle of DMD mice by implantation of primarily cultured MenSCs in vivo. In addition to myogenic differentiation of MenSCs, their cellular fusion with myoblasts was another mechanism of recovery or reacquisition of dystrophin-expressing cells. Ichim et al. reported a patient had suffered Duchenne muscular dystrophy for nearly 20 years before he received treatment of MenSCs, combined with CD34 umbilical cord blood and mixed lymphocyte reaction-matched positive cells [70]. An additive enhancement of muscle strength occurred after two courses of therapy, alongside decreased frequency of respiratory infections, and the reacquisition of general mobility. More encouragingly, these clinical improvements of muscle functioning were maintained for at least 2 years by the time the case was published.

### Respiratory system

Xiang et al. explored the possible benefits of MenSCs in respiratory illness by using a mouse model of acute lung injury (AUI). The mice were treated with MenSCs intravenously and the location of MenSCs in the lungs was detected after only 4 h of injection. MenSC administration significantly repaired the lung impairment by improving pulmonary microvascular permeability, downregulating inflammation, inhibition of apoptosis, and reconstruction of the alveolar-capillary membrane functions. Although preliminary, these results shed a bright light on the future use of MenSCs in the treatment of AUI [21].

## Uncertainty

Although MenSCs may benefit from its complex sub-types of cell components, but the variety in cell sub-population and proportion may also increase the uncertainty of its efficacy. Ex vivo expansion is necessary to produce enough cells for clinical research/therapy; however, standardized MenSC proliferation procedures have not been developed. The presence of donor heterogeneity could affect the biological properties of MenSCs. It has been reported that MenSCs from donors aged over 40 had a diminished long-term proliferation capacity. The long-term in vitro cell culture caused replicative senescence is another potential risk in MenSC study. Senescent non-functional cells may show to experience dramatic changes in terms of gene expression, metabolism, epigenome and, importantly, a distinct secretome profile, known as the senescence-associated secretory phenotype (SASP). The SASP includes pro-inflammatory cytokines as well as chemokines, growth factors, and extracellular matrix-degrading proteins which may influence the activity of surrounding healthy cells [71, 72]. But meanwhile, some studies also demonstrated that though passaging could induce senescence, MenSCs are still able to maintain their normal chromosome karyotypes and normal immunophenotypes up to 20th passage [73]. The development of standardized procedures along with improved culture media and the use of reliable methods, for example, in situ senescence associated with β galactosidase assay and/or a related assay, could allow the identification of the percentage of senescent cells in every samples and contribute to overcoming their adverse effects [71].

## Conclusion

Apart from the practical advantages exemplified by easy accessibility, low cost, minimal ethical hurdles, low immunogenicity, and low tumorgenicity, the therapeutic merits of MenSCs have been to varying extents explored in almost all systems. Plentiful in vivo or in vitro experiments related to a range of conditions with genetic, metabolic, traumatic, and degenerative attributions have shown the same resonance for MenSCs as a fertile source at the service of medical treatment. Besides, many clinical trials have been initiated at various stages with favorable outcomes. The therapeutic effects of MenSCs are not limited to their pluripotency, but extend to their anti-inflammatory action, tropic stimulations to augment endogenous regeneration process, and fusion with other progenitor cells at impaired sites, etc.

Nevertheless, more studies are still required to clarify the following aspects:

- Donor selection: age, timing, etc.
- Standardization of the procedure: While protocols of collection, separation and culture for reliable and consistent acquisition of MenSCs become relatively well established, a consensus on markers with sufficient sensitivity and specificity still needs to be reached.
- A much broader and more comprehensive clinical experience is yet to be collected: safety, duration of effects, long-term impact, etc.

With numerous studies that have embarked on the efficacy of MenSCs, the spectrum of its possible clinical adoption is rapidly widening, and we may be confident to hope our goal of an “off the shelf” strategy for many diseases can be reached.

## Abbreviations

ADSCs: Adipose-derived MSCs
AFMSCs: Amniotic fluid mesenchymal stem cells
ALI: Acute liver injury
AUI: Acute lung injury
CFU-F: Colony-forming unit fibroblasts
CFUs: Colony-forming units
DMD: Duchenne muscular dystrophy
DMEM: Dulbecco’s modified Eagle medium
DMEM/F12: Dulbecco’s Modified Eagle Media: Nutrient Mixture F-12
DPMSCs: Dental pulp-derived stem cells
EDTA: Ethylenediaminetetraacetic acid
eMSCs: Endometrial mesenchymal stem cells
EndoSCs: Endometrial stem cells
ERCs: Endometrial regenerative cells
FGF: Fibroblast growth factor
HLA: Human leukocyte antigen
HLCs: Hepatocyte-like cells
hTERT: Human telomerase reverse transcriptase
IL: Interleukin
IUA: Intrauterine adhesions
LGR5: Leucine-rich repeat-containing G-protein-coupled receptor
MenSCs: Menstrual blood-derived mesenchymal stem cells
MenSCs-Ex: MenSCs-derived exosomes
MI: Myocardial infarction
MSCs: Mesenchymal stem cells
OCT-4: Octamer-binding transcription factor 4
PBS: Phosphate-buffered saline
PDGFR: Platelet-derived growth factor receptor
PH: Partial hepatectomy
POF: Premature ovarian failure
POP: Pelvic organ prolapse
SASP: Senescence-associated secretory phenotype
SCs: Stem cells
Sox2: Sex determining region Y-box2
SP: Side population
SSEA: Stage-specific embryonic antigen
Stro: Stromal cell antigen
SUSD2: Sushi domain containing-2
UCMSCs: Umbilical cord-derived cells MSCs
VSELs: Very small embryonic-like stem cells
